# Whole exome sequencing identifies novel mutation in eight Chinese children with isolated tetralogy of Fallot

**DOI:** 10.18632/oncotarget.22202

**Published:** 2017-10-31

**Authors:** Lin Liu, Hong-Dan Wang, Cun-Ying Cui, Yun-Yun Qin, Tai-Bing Fan, Bang-Tian Peng, Lian-Zhong Zhang, Cheng-Zeng Wang

**Affiliations:** ^1^ Department of Cardiovascular Ultrasound, Henan Provincial People’s Hospital, Zhengzhou University People’s Hospital, Zhengzhou 450003, China; ^2^ Institute of Medical Genetics, Henan Provincial People’s Hospital, Zhengzhou University People’s Hospital, Zhengzhou 450003, China; ^3^ Children’s Heart Center, Henan Provincial People’s Hospital, Zhengzhou University People’s Hospital, Zhengzhou 450003, China; ^4^ Department of Ultrasound, The Affiliated Cancer Hospital, Zhengzhou University, Zhengzhou 450008, China

**Keywords:** tetralogy of Fallot, congenital heart disease, whole exome sequencing

## Abstract

**Background:**

Tetralogy of Fallot is the most common cyanotic congenital heart disease. However, its pathogenesis remains to be clarified. The purpose of this study was to identify the genetic variants in Tetralogy of Fallot by whole exome sequencing.

**Methods:**

Whole exome sequencing was performed among eight small families with Tetralogy of Fallot. Differential single nucleotide polymorphisms and small InDels were found by alignment within families and between families and then were verified by Sanger sequencing. Tetralogy of Fallot-related genes were determined by analysis using Gene Ontology /pathway, Online Mendelian Inheritance in Man, PubMed and other databases.

**Results:**

A total of sixteen differential single nucleotide polymorphisms loci and eight differential small InDels were discovered. The sixteen differential single nucleotide polymorphisms loci were located on Chr 1, 2, 4, 5, 11, 12, 15, 22 and X. Among the sixteen single nucleotide polymorphisms loci, six has not been reported. The eight differential small InDels were located on Chr 2, 4, 9, 12, 17, 19 and X, whereas of the eight differential small InDels, two has not been reported. Analysis using Gene Ontology /pathway, Online Mendelian Inheritance in Man, PubMed and other databases revealed that *PEX5*, *NACA*, *ATXN2*, *CELA1*, *PCDHB4* and *CTBP1* were associated with Tetralogy of Fallot.

**Conclusions:**

Our findings identify *PEX5*, *NACA*, *ATXN2*, *CELA1*, *PCDHB4* and *CTBP1* mutations as underlying genetic causes of isolated tetralogy of Fallot.

## INTRODUCTION

Tetralogy of Fallot (TOF) is the most common cyanotic congenital heart disease (CHD) with an incidence rate of 1/3600 in live births and 10% in CHD [1]. TOF is characterised by cardiac outflow tract malformation caused by non-uniform separation between truncus arteriosus and bulbus arteriosus during embryo stage. TOF pathological features include ventricular septal defect, aortic overriding, right ventricular outflow tract stenosis or pulmonary artery stenosis and right ventricular hypertrophy. In neonatal period, children with TOF may manifest oxygen deficiency, pneumonia, intractable congestive heart failure and other complications with high early mortality. Despite TOF treatment in children, some complications will still occur because of poor prognosis. TOF brings heavy burden to the family and society. Therefore, to investigate TOF etiology, possible pathogenesis and risk factors for prenatal diagnosis and counselling and prognostic evaluation is of great significance.

Heart development is a complex and orderly process including cardiac tube formation, loop formation, intracardiac separation and vascular connection. Heart development is related to many genes, which express and interact in different spaces and at different times to form a precise regulatory mechanism. Abnormal expression occurring in any of these genes is likely to affect heart development, leading to heart malformation. For TOF, embryonic developmental mechanism is nearly definite. However, the molecular pathogenesis remains to be clarified. TOF is associated with gene mutation [2]. TOF-related genes include *NKX2-5* [3], *GATA4* [3, 4], and *JAG-1* [5, 6]. Approximately 15% of TOF are from 22q11 microdeletion syndrome [7] caused by chromosome 22 long arm 1 zone 1 subzone deletion and manifest extracardiac abnormalities, such as abnormal face, thymic hypoplasia, cleft palate and hypocalcemia. In addition, TOF is also related to chromosomal aneuploidy, and approximately 3% of TOF is from 21-trisomy syndrome [8]. However, the pathogenesis of isolated TOF is still to be elucidated.

Whole exome sequencing (WES) is suitable to high-throughput sequencing for all genomic exon regions. Human exome accounting for approximately 1%–2% of genome contains important information of protein synthesis, which directly reflects gene function. In most diseases, related mutations are located in the exome region. WES may be used to find pathogenic gene and predisposing gene in complex diseases, monogenic diseases and cancer because it can investigate protein encoding information in several individuals; its data are also accurate [9]. The aim of this study was to find TOF-related pathogenic genes through WES technology performed among eight small families with TOF, providing a basis for studying TOF pathogenesis.

## RESULTS

### Clinical features

**Figure 1:**
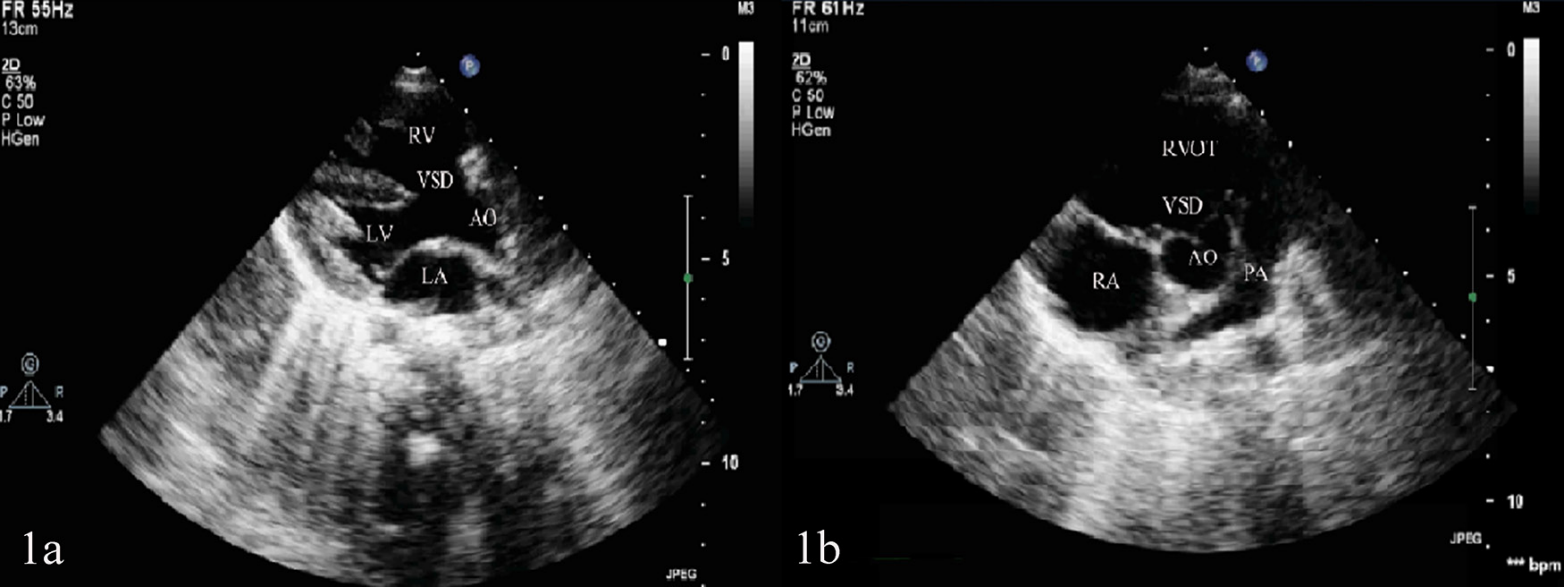
**(A)** Ventricular septal defect and aortic overriding shown in the left ventricular long-axis view of transthoracic echocardiography. **(B)** Pulmonary artery stenosis shown in the aorta short-axis view of transthoracic echocardiography. LA: left atrium; LV: left ventricle; AO: aorta; VSD: ventricular septal defect; RA: right atrium; RV: right ventricle; PA: pulmonary artery; RVOT: right ventricular outflow tract.

All the eight TOF children manifested cyanosis and rough systolic-ejection murmurs at the left sternal border between the second and fourth ribs. Echocardiography showed right ventricular enlargement, right ventricular anterior wall thickening, right ventricular outflow tract and pulmonary artery stenosis, ventricular septal defect and aortic overriding (Figure 1). The echocardiographic results of eight TOF children in the right ventricle, right ventricular anterior wall, right ventricular outflow tract, main pulmonary artery, left pulmonary artery, right pulmonary artery, ventricular septal defect and left ventricular ejection fraction (Table 1). The eight TOF children displayed no other malformations, and their parents showed no abnormalities.

**Table 1:** Results of echocardiogram in the 8 children with tetralogy of Fallot

ID	Gender	Age	RV (mm)	RVAW (mm)	RVOT (mm)	MPA (mm)	LPA (mm)	RPA (mm)	VSD (mm)	LVEF (%)
1	female	3months	8.0	5.0	5.4	5.6	4.3	4.8	9.5	73
2	male	8months27days	12.0	6.0	5.7	5.3	4.3	5.3	10.2	68
3	male	1years11months	13.9	6.8	7.2	10.5	6.6	6.7	13.5	72
4	female	2months24days	11.8	4.1	6.0	15.3	9.3	7.7	11.5	57
5	female	6months	11.1	6.1	9.0	11.8	7.6	7.6	12.9	67
6	male	8months2days	13.0	6.3	7.0	6.4	4.2	4.4	13.5	72
7	female	11months26days	13.4	5.9	5.8	12.4	6.7	7.1	13.1	69
8	male	6months11days	10.3	5.0	6.1	9.0	4.3	5.7	13.2	74

RV: right ventricle; RVAW: right ventricular anterior wall; RVOT: Right ventricular outflow tract; MPA: main pulmonary artery; LPA: left pulmonary artery; RPA: right pulmonary artery; VSD: ventricular septal defect; LVEF: left ventricular ejection fraction.

### Mutation detection

A total of sixteen differential single nucleotide polymorphisms (SNPs) loci and eight differentially small InDels were discovered by sequencing all exons in the eight small families and alignment of SNPs and small InDels within families and between families (Table 2 and Table 3).

**Table 2:** SNPs identified by whole exome sequencing

Chr	Position	ID	Ref	Alt	Mutation	Symbol	Amino acids	SIFT	PolyPhen
chr12	56717682	rs745387506	T	G	missense	*NACA*	H/P	-	unknown(0)
chr5	141123595	-	T	A	missense	*PCDHB4*	S/T	tolerated (1)	benign(0)
chr12	7190555	-	G	C	missense	*PEX5*	A/P	tolerated (0.22)	benign(0.067)
chr2	240042663	-	C	T	missense	*PRR21*	R/H	tolerated (0.56)	unknown(0)
chr4	1237208	-	A	T	stop_gained	*CTBP1*	C/*	-	-
chrX	37010556	rs61999275	G	C	missense	*FAM47C*	A/P	tolerated(1)	benign(0)
chr1	26282352	rs6667693	C	A	missense	*UBXN11*	G/C	tolerated (0.05)	unknown(0)
chr1	86368686	rs75376884	T	C	missense	*ODF2L*	M/V	tolerated (0.14)	benign(0.001)
chr4	87614868	-	G	A	missense	*DSPP*	D/N	-	unknown(0)
chr4	87614877	rs150132251	A	G	missense	*DSPP*	N/D	-	unknown(0)
chr4	88008301	rs117078377	G	A	missense	*PKD2*	A/T	tolerated(0.3)	benign(0.395)
chr11	71527593	rs199903176	A	G	missense	*KRTAP5-7*	Y/C	tolerated (0.22)	unknown(0)
chr12	51346625	rs117443541	T	G	missense	*CELA1*	Y/S	tolerated(0.13)	benign(0.045)
chr15	88856792	rs12899191	A	G	missense	*ACAN*	T/A	tolerated(0.56)	benign(0.084)
chrX	104250509	rs9697856	T	G	missense	*ESX1*	T/P	tolerated (0.54)	unknown(0)
chr22	11068057	-	A	T	missense	*BAGE5*	R/W	tolerated (0.12)	damaging(0.675)

Chr: chromosome; Ref: reference sequence base; Alt: alternative base identified; SIFT: sorting intolerant from tolerant.

**Table 3:** Small InDels identified by whole exome sequencing

Chr	Position	ID	Ref	Alt	Mutation	Symbol	Amino acids	SIFT	PolyPhen
chr2	104856098	rs775150602	AGCC	A	inframe_deletion	*POU3F3*	A/-	-	-
chr12	111598972	rs769170503	C	CTGT	inframe_insertion	*ATXN2*	Q/QQ	-	-
chr4	13632182	.	CT	C	splice_acceptor_variant&non_coding_transcript_variant	*-*	-		
chr9	136327352	.	ACCCCCTC	A	TF_binding_site_variant&TFBS_ablation	*GPSM1*	-	-	-
chr17	75616380	rs371699907	CAGG	C	inframe_deletion	*MYO15B*	QE/Q	-	-
chr19	9251064	rs201985790	CT	C	frameshift_variant	*OR7E24*	F/X	-	-
chr19	51694076	rs10689461	C	CAG	splice_acceptor_variant&non_coding_transcript_variant	*SPACA6P*	-	-	-
chrX	19345745	rs776856509	TC	TCC, T	frameshift_variant	*PDHA1*	S/X	-	-

Ten of the sixteen differential SNPs were reported, namely, Chr1 (rs6667693, *UBXN11*), Chr1 (rs75376884, *ODF2L*), Chr4 (rs150132251, *DSPP*), Chr4 (rs117078377, *PKD2*), Chr11 (rs199903176, *KRTAP5-7*), Chr12 (rs745387506, *NACA*), Chr12 (rs117443541, *CELA1*), Chr15 (rs12899191, *ACAN*), ChrX (rs61999275, *FAM47C*) and ChrX rs9697856, *ESX1*). Six of the 16 differential SNPs were not reported, namely, Chr 2 (*PRR21*), Chr 4 (*CTBP1*), Chr 4 (*DSPP*), Chr 5 (*PCDHB4*), Chr12 (*PEX5*) and Chr22 (*BAGE5*).

Six of the eight differentially small InDels were reported, namely, Chr2 (rs775150602, *POU3F3*), Chr12 (rs769170503, *ATXN2*), Chr17 (rs371699907, *MYO15B*), Chr19 (rs201985790, *OR7E24*), Chr19 (rs10689461, *SPACA6P*) and ChrX (rs776856509, *PDHA1*). Two of the eight differentially small InDels were not reported, namely, Chr4 (position: 13632182) and Chr9 (*GPSM1*).

SNPs and small InDel-related Online Mendelian Inheritance in Man (OMIM) genes and clinical phenotypes are shown in Table 4.

**Table 4:** SNPs- and small InDels-related OMIM genes and clinical phenotypes

Gene	Chromosome	OMIM	Gene description	Function
*NACA*	Chr12	601234	gene encodes a protein that associates with *BTF3* to form *NAC*	skeletal development
*PCDHB4*	Chr5	606330	a member of the protocadherin beta gene cluster	cell-cell neural connections
*PEX5*	Chr12	600414	peroxisomal biogenesis factor 5	Peroxisome biogenesis disorder 2A, 2B; Rhizomelic chondrodysplasia punctata, type 5
*PRR21*	Chr2	-	-	-
*CTBP1*	Chr4	602618	gene encodes a protein that binds to the C-terminus of adenovirus E1A proteins	transcriptional repressor and cellular proliferation
*FAM47C*	ChrX	-	-	-
*UBXN11*	Chr1	609151	a protein with a divergent C-terminal UBX domain	affect the actin cytoskeleton and alter cell shape
*ODF2L*	Chr1	-	-	-
*DSPP*	Chr4	125485	a member of the small integrin-binding ligand N-linked glycoprotein family of proteins	Deafness, autosomal dominant 39, with dentinogenesis; Dentin dysplasia, type II; Dentinogenesis imperfecta, Shields type II, III
*PKD2*	Chr4	173910	a member of the polycystin protein family	Polycystic kidney disease 2
*KRTAP5-7*	Chr11	-	-	-
*CELA1*	Chr12	130120	Elastases form a subfamily of serine proteases that hydrolyze many proteins in addition to elastin	evolutionarily silenced in pancreatic acinar cells
*ACAN*	Chr15	155760	a member of the aggrecan/versican proteoglycan family	Osteochondritis dissecans, short stature, and early-onset osteoarthritis; Spondyloepimetaphyseal dysplasia, aggrecan type; Spondyloepiphyseal dysplasia, Kimberley type
*ESX1*	ChrX	300154	gene encodes a dual-function 65 kDa protein that undergoes proteolytic cleavage to produce a 45 kDa N-terminal fragment with a paired-like homeodomain and a 20 kDa C-terminal fragment with a proline-rich domain	placental development and spermatogenesis
*BAGE5*	Chr22	-	-	-
*POU3F3*	Chr2	602480	a member of the class III *POU* family of transcription factors	expressed in the central nervous system
*ATXN2*	Chr12	601517	gene belongs to a group of genes that is associated with microsatellite-expansion diseases, a class of neurological and neuromuscular disorders caused by expansion of short stretches of repetitive DNA	Spinocerebellar ataxia 2; susceptibility to Amyotrophic lateral sclerosis; susceptibility to Parkinson disease, late-onset
*GPSM1*	Chr9	609491	gene encodes a receptor-independent activator of G protein signaling,	influence the basal activity of G-protein signaling systems
*MYO15B*	Chr17	-	-	-
*OR7E24*	Chr19	-	-	-
*SPACA6P*	Chr19	-	-	-
*PDHA1*	ChrX	300502	a nuclear-encoded mitochondrial multienzyme complex that catalyzes the overall conversion of pyruvate to acetyl-CoA and CO(2), and provides the primary link between glycolysis and the tricarboxylic acid cycle	Pyruvate dehydrogenase E1-alpha deficiency

We discovered fourteen SNPs- and Small InDels-related OMIM genes, including: *NACA*, *PCDHB4*, *PEX5*, *CTBP1*, *UBXN11*, *DSPP*, *PKD2*, *CELA1*, *ACAN*, *ESX1*, *POU3F3*, *ATXN2*, *GPSM1*, *PDHA1. PEX5* (OMIM:600414) is associated with Peroxisome biogenesis disorder 2A, 2B. *NACA* (OMIM:601234) is associated with skeletal development. *ATXN2* (OMIM:601517) is associated with Spinocerebellar ataxia 2, susceptibility to Amyotrophic lateral sclerosis, susceptibility to Parkinson disease and late-onset. *CELA1* (OMIM:130120) is associated with evolutionarily silenced in pancreatic acinar cells. *PCDHB4* (OMIM:606330) cell-cell neural connections is associated with *CTBP1*(OMIM:602618) transcriptional repressor and cellular proliferation.

### Results verified by Sanger sequencing

**Figure 2:**
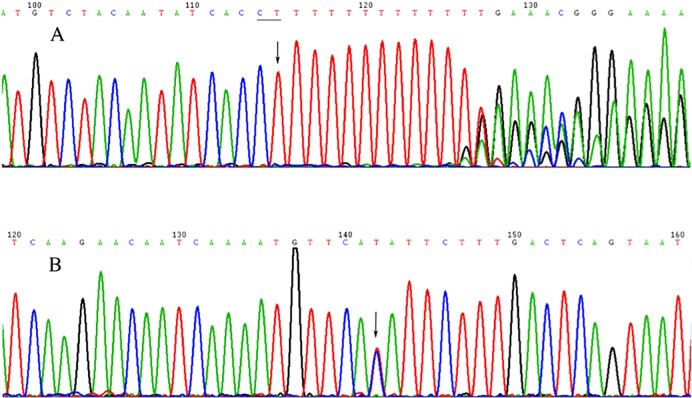
Diagram of the Sanger sequencing for two mutations **(A)** chr4:13632182 (CT→C). **(B)** chr1:86368686 (T→C).

We designed twenty-four pairs of primers to verify sixteen differential SNPs loci and eight differentially small InDels. The results of Sanger sequencing are consistent with that of WES (Figure 2).

The verification of sixteen differential SNPs sites included *NACA* (rs745387506, T>G), *FAM47C* (rs61999275, G>C), *UBXN11* (rs6667693, C>A), *ODF2L* (rs75376884, T>C), *DSPP* (rs150132251, A>G), *PKD2* (rs117078377, G>A), *KRTAP5-7* (rs199903176, A>G), *CELA1* (rs117443541, T>G), *ACAN* (rs12899191, A>G), *ESX1* (rs9697856, T>G), *PCDHB4* (chr5, 141123595, T>G), *PEX5* (chr12, 7190555, G>C), *PRR21* (chr2, 240042663, C>T), *CTBP1* (chr4, 1237208, A>T), *DSPP* (chr4, 87614868, G>A) and *BAGE5* (chr22, 11068057, A>T) were investigated.

The verification of eight differentially small InDels sites included *AGCC* (rs775150602, AGCC>A), *ATXN2* (rs769170503, C>CTGT), *MYO15B* (rs371699907, CAGG>C), *OR7E24* (rs201985790, CT>C), *SPACA6P* (rs10689461, C>CAG), *PDHA1* (rs776856509, TC>TCCT), *GPSM1* (chr9, 136327352, ACCCCCTC>A), and (chr4, 13632182, CT>T) were investigated.

### Gene ontology (GO)/pathway analysis

**Figure 3:**
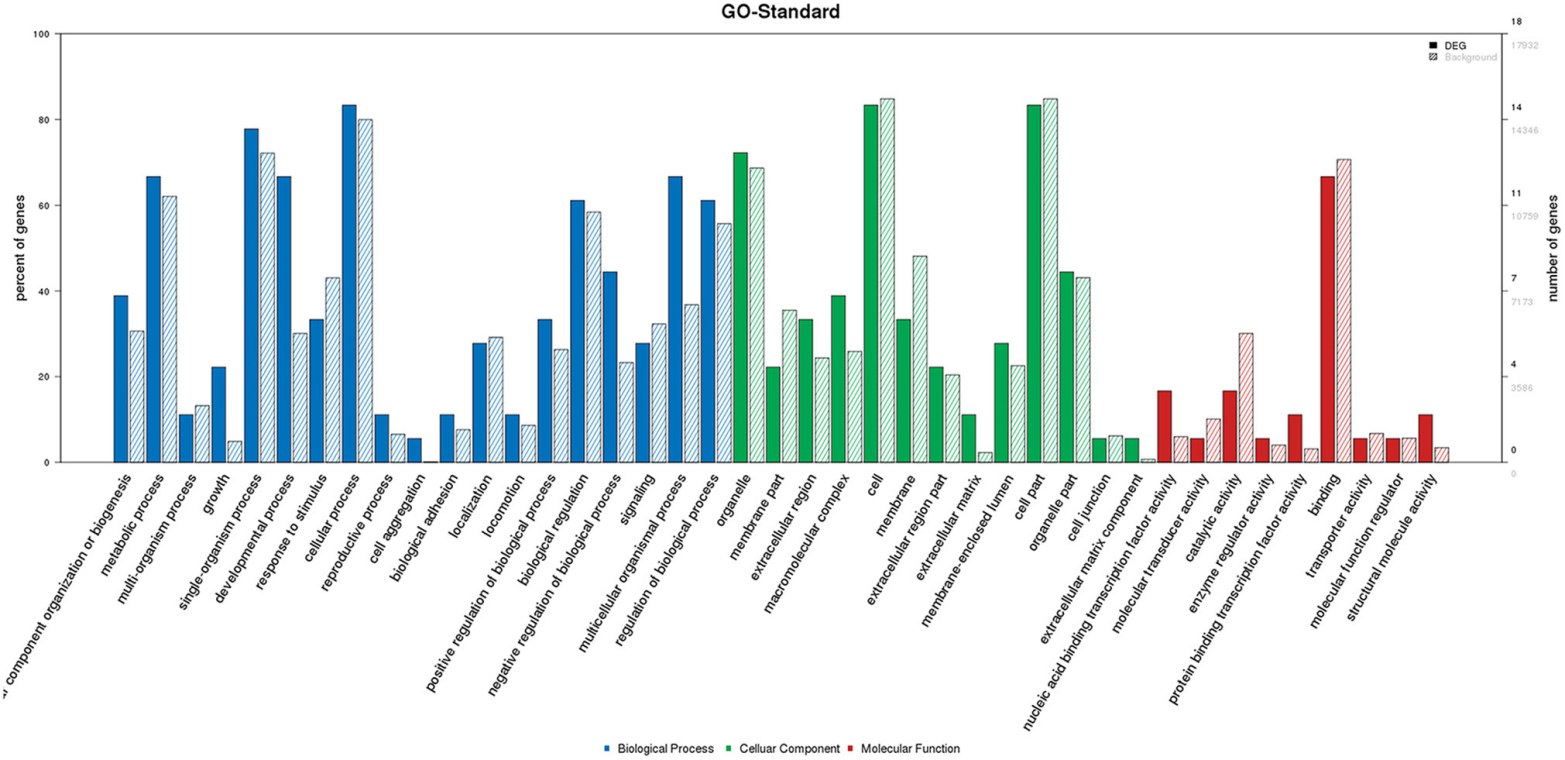
GO analysis of 22 genes

GO analysis indicated the percentages of 22 genes enriched in GO term (Figure 3). Growth was related to CHD, and it was associated with some genes including *PEX5*, *NACA*, *ATXN2* and *CELA1* (Table 5). *PEX5*, *ATXN2* and *CELA1* are responsible for multicellular organism growth (GO:0035264). *PEX5*, *ATXN2*, *NACA* and *CELA1* are responsible for developmental growth (GO:0048589). *PEX5*, *ATXN2* and *NACA* are responsible for regulation of developmental growth (GO:0048638) and growth regulation (GO:0040008). *PEX5* and *ATXN2* are responsible for regulation of multicellular organism growth (GO:0040014). *PEX5* and *NACA* are responsible for positive regulation of developmental growth (GO:0048639) and positive growth regulation (GO:0045927). *PEX5* is responsible for positive regulation of multicellular organism growth (GO:0040018). *ATXN2* is responsible for negative regulation of multicellular organism growth (GO:0040015), epidermal growth-factor receptor binding (GO:0005154), negative regulation of developmental growth (GO:0048640), growth-factor receptor binding (GO:0070851) and negative growth regulation (GO:0045926).

**Table 5:** Genes involved by growth and possibly associated with CHD in GO term

Gene	Term	Database	ID
*PEX5*:5830|*ATXN2*:6311|*CELA1*:1990	multicellular organism growth	Biological Process	GO:0035264
*PEX5*:5830|*ATXN2*:6311|*NACA*:4666|*CELA1*:1990	developmental growth	Biological Process	GO:0048589
*PEX5*:5830|*ATXN2*:6311|*NACA*:4666	regulation of developmental growth	Biological Process	GO:0048638
*PEX5*:5830|*ATXN2*:6311	regulation of multicellular organism growth	Biological Process	GO:0040014
*PEX5*:5830|*NACA*:4666	positive regulation of developmental growth	Biological Process	GO:0048639
*PEX5*:5830|*ATXN2*:6311|*NACA*:4666|*CELA1*:1990	growth	Biological Process	GO:0040007
*PEX5*:5830|*NACA*:4666	positive regulation of growth	Biological Process	GO:0045927
*PEX5*:5830|*ATXN2*:6311|*NACA*:4666	regulation of growth	Biological Process	GO:0040008
*PEX5*:5830	positive regulation of multicellular organism growth	Biological Process	GO:0040018
*ATXN2*:6311	negative regulation of multicellular organism growth	Biological Process	GO:0040015
*ATXN2*:6311	epidermal growth factor receptor binding	Molecular Function	GO:0005154
*ATXN2*:6311	negative regulation of developmental growth	Biological Process	GO:0048640
*ATXN2*:6311	growth factor receptor binding	Molecular Function	GO:0070851
*ATXN2*:6311	negative regulation of growth	Biological Process	GO:0045926

**Figure 4:**
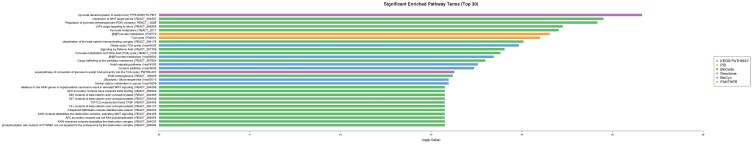
Pathway analysis of 22 genes

Repression of Wnt target genes and Notch signalling were related to CHD, and was associated with *PCDHB4* and *CTBP1* genes (Table 6). Pathway analysis showed that twenty-two genes were enriched at the top fifty of pathway term (Figure 4). Genes involved in the Wnt (*CTBP1* and *PCDHB4*) and Notch (*CTBP1*) signalling are important for pathway analysis in cardiac development. The mutation sites in the six genes were as follows: *PEX5* (A60P), *NACA* (H1283P), *ATXN2* (Q21QQ), *CELA1* (Y5S), *PCDHB4* (S533T) and *CTBP1* (C35^*^). The transcript number of *ATXN2* (Q21QQ) is ENST00000608853.5, and the transcript number of CTBP1 (C35^*^) is ENST00000629223.1.

**Table 6:** Genes involved by pathway and possibly associated with CHD

Gene	Term	Database	ID
*CTBP1*:1487	repression of WNT target genes	Reactome	REACT_264567
*CTBP1*:1487	*AXIN* mutants destabilize the destruction complex, activating WNT signaling	Reactome	REACT_264496
*CTBP1*:1487	deletions in the *AXIN* genes in hepatocellular carcinoma result in elevated WNT signaling	Reactome	REACT_264286
*CTBP1*:1487	WNT signaling pathway	KEGG PATHWAY	hsa04310
*CTBP1*:1487	*TCF* dependent signaling in response to WNT	Reactome	REACT_264596
*CTBP1*:1487	*RNF* mutants show enhanced WNT signaling and proliferation	Reactome	REACT_264378
*CTBP1*:1487	Signaling by WNT	Reactome	REACT_11045
*CTBP1*:1487	Notch signaling pathway	KEGG PATHWAY	hsa04330
*PCDHB4*:56131|*CTBP1*:1487	WNT signaling pathway	PANTHER	P00057

### Variant analysis

**Figure 5:**
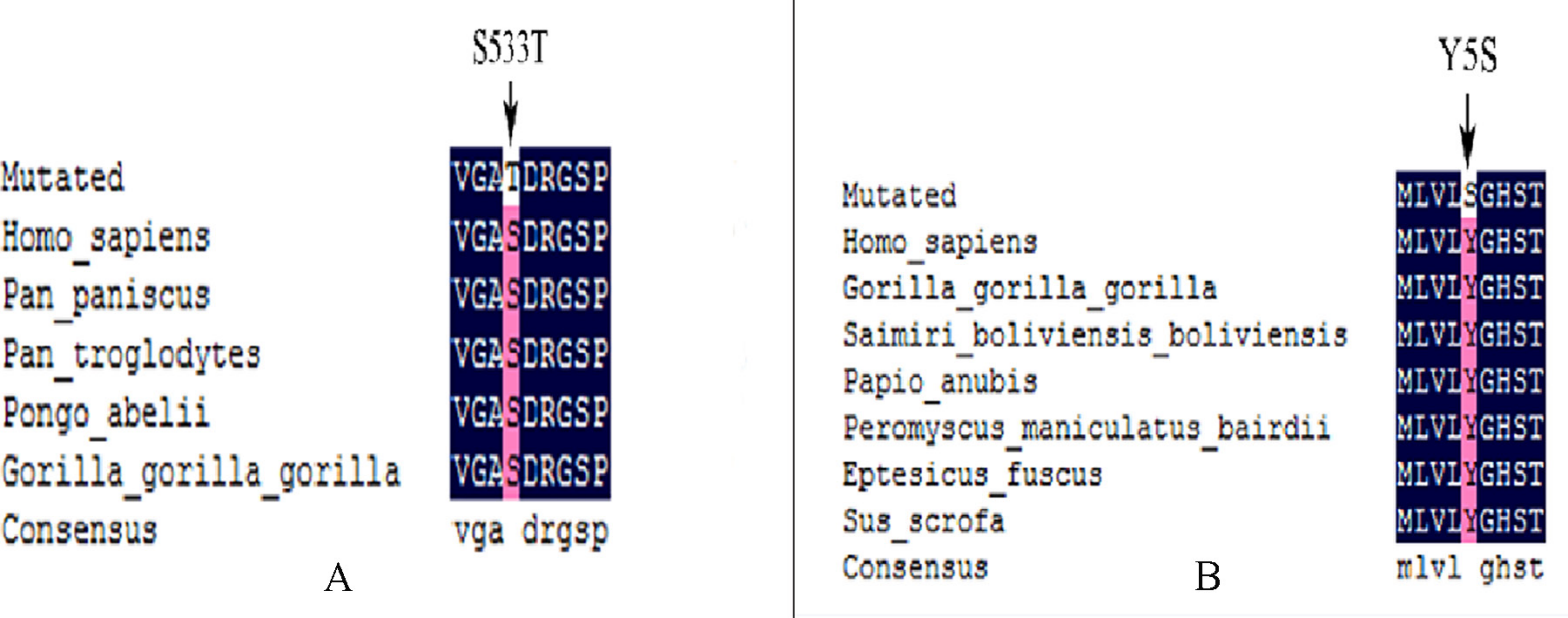
**(A)** Alignment of multiple *PCDHB4* protein sequences in different species reveals that the S533T amino acid is located in the highly conserved amino acid region in different species. **(B)** Alignment of multiple *CELA1* protein sequences in different species reveals that the Y33T amino acid is located in the highly conserved amino acid region in different species.

By alignment of protein sequences of *PEX5*, *NACA*, *ATXN2*, *CELA1*, *PCDHB4* and *CTBP1*, S5333T of *PCDHB4* was highly evolutionarily conserved in diverse species including *Homo sapiens*, *Pan paniscus*, *Pongo abelii* and gorilla (Figure 5a). In addition, Y5S of *CELA1* was highly evolutionarily conserved in diverse species including *H. sapiens*, gorilla, Saimiri *boliviensis*, *Papio anubis*, *Peromyscus maniculatus*, *Tapirus bairdii*, *Eptesicus fuscus* and *Sus scrofa* (Figure 5b). The highly evolutionarily conserved protein sequences were not found in *PEX5*, *NACA*, *ATXN2* and *CTBP1* in other species.

## DISCUSSION

TOF as the most common cyanotic CHD is characterised by right ventricular outflow tract obstruction, pulmonary stenosis, ventricular septal defect and aortic overriding caused by non-uniform separation between the truncus arteriosus and bulbus arteriosus at embryo stage. Currently, surgical repair is usually performed in the first month after birth to relieve the stenosis of the right outflow tract and close ventricular septal defect, enabling an exclusive ejection of oxygenated blood via the left ventricle. Cumulative survival and event-free survival were 72% and 25%, respectively, 40 years after follow-up [12].

TOF etiology still needs to be completely clarified because of TOF complexity attributed by both genetic and nongenetic effectors. Next-generation sequencing technology, such as WES with rapid, high throughput and cost-effective features, can meet the requirements for medical studies. WES has been used to obtain information about genetic alterations and potential predispositions possibly associated with TOF occurrence.

Eight small families were tested through WES, and differential SNP loci and InDels were found. From the comparison within each family and among the families, sixteen differential SNPs loci and eight differential Small InDels were screened out. Corresponding genes exist in the database, wherein ten of the sixteen differential SNPs have been reported and six of the sixteen differential SNPs have not been reported. Among the eight Small InDels, seven small Indels have corresponding genes in the database and one Small InDels does not have. Six of the eight differential Small InDels have been reported, whereas two of them have not. Among them, no gene in the database corresponds to the mutation of chr4, position: 13632182. It might be located among the genes or among noncoding regions, and the function requires further study. Go/Pathway analysis of the sixteen differential SNPs loci and eight differential Small InDels were conducted and *PEX5*, *NACA*, *ATXN2*, *CELA1*, *PCDHB4* and *CTBP1* were finally screened out and were associated with TOF.

Similar to some genes found to be related to TOF by detection of chromosome karyotype, gene copy number variation and de novo mutation (Table 7), Greenway et al. [13] found four genes (*PRKAB2*, *CHD1L*, *BCL9* and *GJA5*) with the highest expression in the right ventricular outflow tract, which is malformed in TOF. Bittel et al. [14] included both idiopathic TOF patients and three patients, which harbour the syndromic 22q11.2 deletion. The expression of the genes *A2BP1*, *VEGF*, *NPPA* and *NPPB* located in the 22q11.2 region was half-reduced among syndromic patients as expected, whereas none of these genes was differentially expressed in any of the idiopathic TOF subjects. Notch pathway was also suppressed in patients with 22q11.2 deletions. Sharma et al. [15, 16] found that TOF expression of the majority of the genes associated with Wnt and Notch signalling pathways was significantly reduced. Their results also confirmed an upregulated expression of *VEGF* and proteins of the extracellular matrix reported in previous studies. Feiner et al. [17] found a novel locus at 1q32.2 in two unrelated TOF who displayed loss of copy number variations overlapping the *PLXNA2* gene. Knockout mice deficient for *PLXNA2* display CHD including TOF [18]. Amarillo et al. [19] found a 3.76 Mb de novo contiguous gain of 9q34.2-q34.3 by chromosome microarray analysis and then also confirmed it by karyotype analysis and fluorescence *in situ* hybridization. This duplicated interval disrupted retinoid X receptor alpha (OMIM ^#^180245) at intron 1. To our knowledge, *PEX5*, *PCDHB4*, *CTBP1*, *NACA*, *ATXN2* and *CELA1* are novel SNPs associated with TOF, which have not been reported.

**Table 7:** Summary of genes with a potential relevance to TOF

References	Subjects	Platform	Genes	Conclusions
Steven C et al (2009)	114 TOF Trios 398 TOF	Affymetrix Human Genome Wide SNP Array 6.0	*PRKAB2, CHDIL, BCL9, GJA5*	potential TOF candidate gene
Douglas C Bittel et al (2011)	19 TOF	Applied Microarrays Inc. CodeLink Human Whole Genome Bioarray	*A2BP1, VEGF, NPPA, NPPB*,	potential relevance for TOF
Amy Rodemoyer et al (2011)	16 TOF	Affymetrix Human Exon 1.0 ST v2	*NEFH, SST, TF, KRT6A, KRT7, NTRK2, MYL2, PPBP, NRGN*, *APOC3*	dysregulated network in TOF
Goodship et al (2012)	362 TOF	SEQUENOM MALDI-TOF	*PTPN11*	risk allele for TOF
Candice K et al (2012)	433 TOF	Affymetrix Genome-Wide Human SNP Array 6.0	*GJA5, PLXNA2*	potential TOF candidate gene
Rachel Soemedi et al (2012)	283 TOF Trios	Illumina 660W SNP Array 6.0	*GJA5, HAND2, EDIL3, CNOT6*	potential TOF candidate gene
Heather J.Cordell et al (2013)	798 TOF	Illumina 600W QUAD array	*PTPN11, GPC5, NRP1*	risk allele for TOF
Marcel Grunert et al (2014)	22 TOF	NimbleGen sequence capture 365K array Genome Sequencer FLX Illumina Genome Analyzer	deleterious SNPs in *BARX1, BCCIP, DAG1, EDN1, FANCL, FANCM, FMR1, FOXK1, HCN2, MYOM2, PEX6, ROCK1, TCEB3, TP53BP2, TTN, WBSCR16*	imbalance of functional networks in TOF
Tan ZP et al (2015)	1 TOF	Illumina Hiseq2000	*SCN5A*	a possible cause of TOF
LaHaye S et al (2016)	2 TOF	Illumina Hiseq2000	*MYBPC3, SOS1*	uncertain significance

Our study aimed to identify other monogenic causes of TOF. GO analysis indicated that *PEX5* was related to multicellular organism growth (GO:0035264), developmental growth (GO:0048589), regulation of developmental growth (GO:0048638), regulation of multicellular organism growth (GO:0040014), positive regulation of developmental growth (GO:0048639), positive regulation of growth (GO:0045927), growth (GO:0040007) and positive regulation of multicellular organism growth. In the 26 cases with TOF, Grunert M et al. [20] found deleterious SNPs in 16 genes, including *BARX1*, *BCCIP, DAG1*, *EDN1*, *FANCL*, *FANCM*, *FMR1*, *FOXK1*, *HCN2*, *MYOM2*, *PEX6*, *ROCK1*, *TCEB3*, *TP53BP2*, *TTN* and *WBSCR16* which resulted in the imbalance of function networks in TOF. Huffnagel IC et al.[21] found that two of the 21 genetically proven Rhizomelic chondrodysplasia punctata patients suffered from TOF and showed *PEX7* mutations. Plasmalogens were not detected in cardiac tissue of *PEX7* knock-out mice, which is a model for RCDP type 1. The above-mentioned *PEX5, PEX6* and *PEX7* are all *PEX* family gene named Peroxins (PEXs) which are proteins essential for the assembly of functional peroxisomes. Therefore, *PEX5* might participate in TOF growth and development process. *PCDHB4* is located on Chr5q31.3. This gene is a member of the protocadherin beta gene cluster and is one of the three related and tandemly linked gene clusters at chromosome 5. Their specific functions are unknown but they most likely play a critical role in the establishment and function of specific cell–cell neural connections. Alazami AM et al. [22] performed WES on 143 multiplex consanguineous families, in which known disease genes had been excluded by autozygosity mapping. Patients, whose clinical phenotype is featured by global developmental delay and brain atrophy, have mutation in *PCDHB4* (NM_021908:c.489T>G:p.Y163X) based on the candidate gene analysis, and the function of which participated in the growth and development process. Base on our research, Go analysis indicated that *PCDHB4* (P00057) was related to WNT signaling pathway. Thus, *PCDHB4* (P00057) may be a potential TOF candidate gene. *NACA* is located on Chr12q13.3. The protein encoded by *NACA* is associated with basic transcription factor 3, which forms the nascent polypeptide-associated complex. In this study, GO analysis indicated that *NACA* was related to positive regulation of developmental growth (GO:0048639) and positive regulation of growth (GO:0045927). Thus, *NACA* may be a potential TOF candidate gene. *CELA1* is located on Chr12q13.13. This gene forms a subfamily of serine proteases that hydrolyze many proteins in addition to elastin. Humans possess six elastase genes, which encode the structurally similar elastases 1, 2, 2A, 2B, 3A and 3B. Unlike other elastases, pancreatic elastase 1 is not expressed in the pancreas. GO analysis indicated that *CELA1* was related to multicellular organism growth (GO:0035264), developmental growth (GO:0048589) and growth (GO:0040007). Thus, *CELA1* may have a potential relevance to TOF. *ATXN2* is located on Chr12q24.12 with a transcript number of ENST00000608853.5. This gene belongs to a group of genes that are associated with microsatellite-expansion diseases, a class of neurological and neuromuscular disorders caused by expansion of short stretches of repetitive deoxyribonucleic acid (DNA). Genome-wide association studies indicate that loss-of-function mutations in this gene may be associated with susceptibility to type I diabetes, obesity and hypertension. Alternative splicing leads to multiple transcript variants. GO analysis indicated that *ATXN2* was related to negative regulation of multicellular organism growth (GO:0040015), epidermal growth-factor receptor binding (GO:005154), negative regulation of developmental growth (GO:0048640), growth-factor receptor binding (GO:0070851) and negative regulation of growth (GO:0045926). Thus, *ATXN2* may have a potential relevance to TOF. *CTBP1* is located on Chr4p16.12 with a transcript number of ENST00000629223.1. This gene encodes a protein that binds to the C-terminus of adenovirus E1A proteins. This phosphoprotein is a transcriptional repressor and may play a role in cellular proliferation. This protein and the product of a second closely related gene, *CTBP1*, can dimerise. Both proteins can also interact with a polycomb group protein complex, which participates in regulation of gene expression during development. Alternative splicing of transcripts from this gene results in multiple transcript variants. GO analysis indicated that *CTBP1* was related to Wnt signalling pathway and Notch signalling pathway. Thus, *CTBP1* may be a potential TOF candidate gene.

This study has some limitations, and the sample size is small. In the future, the number of samples will be further increased and an in-depth research will be conducted. The identified mutation sites have not been verified based on large TOF sample (n>50). Thus, whether the identified mutation sites may occur in a large group of TOF people has not been confirmed. Moreover, further study is necessary to verify relevant function genes and accordingly illustrate the pathogenesis of TOF.

In summary, TOF pathogenesis is complex because TOF is associated with more than one genetic variant. Eight small families were observed through WES and found that TOF-related genes were *PEX5, NACA, ATXN2, CELA1, PCDHB4 and CTBP1*, whose potential function might participate in TOF growth and development process. Although none of the three variants were predicted to be highly influential, they are in genes belonging to networks involved in relevant developmental pathways. Further study on the potential developmental mechanisms will be conducted based on these results.

## MATERIALS AND METHODS

### Subjects

A total of eight small families whose Chinese children received surgical TOF treatment at the Children’s Heart Center of Henan Provincial People’s Hospital between January 2016 and June 2016 were enrolled in this study. The eight children ageing 3–11 months included four male babies and four female babies. All manifested isolated TOF. Their parents were normal. All the eight children were diagnosed with TOF by echocardiogram, clinical symptoms and signs and intraoperative findings.

### Methods

#### DNA extraction

In each member of the eight small families including 24 individuals, 600 μl peripheral blood was collected to extract genomic DNA using genomic DNA Extraction Kit (Qiage, Germany). The remaining blood samples were stored at −80°C.

#### WES and mutation screening

WES and subsequent variant annotation were performed on genomic DNA derived from the eight TOF children and their parents. Paired-end libraries were prepared according to the manufacturer’s protocols (Agilent). Exome libraries for the eight families were constructed using Agilent SureSelectXT Target Enrichment System according to Illumina Paired End Sequencing Protocol (Agilent Technologies, CA, USA). Capturing of whole exon was carried out according to the protocol of Agilent’s Sure Select Human All 1 UTRs 71 MB v4 kit. The flow cells were sequenced as paired-end 150 base pair reads on an Illumina HiSeq ×10 platform to a minimum depth of 50× targeted region coverage using TruSeq SBS sequencing kit version 3 and HiSeq data collection version 2.0.12.0 software (Illumina, Inc., San Diego, CA, USA).

The raw sequence reads we obtained were aligned to the human genome reference sequence (hg19) using Burrows–Wheeler Aligner (BWA) with standard parameters [10]. The BWA-aligned reads were statistically calculated using PICARD software to exclude polymerase chain reaction duplicates. Regional realignment and quality score recalibration were carried out using Genome Analysis Toolkit [11] with recommended parameters, which included local realignment of the sequences around InDels, base quality score recalibration, variant calling and variant quality score recalibration.

#### Variant detection in the eight TOF children by Sanger sequencing

BigDye^®^ Terminator v3.1 Cycle Sequencing Kit (Applied Biosystems, Foster City, CA, USA) and ABI 3130 Genetic Analyzer were used to detect variants among the eight TOF children.

### Ethics statement

Approval was obtained from local ethics committees of Henan Provincial People’s Hospital, Zhengzhou University People’s Hospital. Informed consents were provided to all the participants of this study.

## Abbreviations

TOF: tetralogy of Fallot
CHD: congenital heart disease
WES: whole-exome sequencing
SNPs: single nucleotide polymorphisms
CNVs: copy number variations
DNA: deoxyribonucleic acid
BWA: Burrows–Wheeler Aligner
GATK: Genome Analysis Toolkit
BTF3: basic transcription factor 3
NAC: nascent polypeptide-associated complex
SRP: signal recognition particle
